# Cluster of SARS among Medical Students Exposed to Single Patient, Hong Kong

**DOI:** 10.3201/eid1002.030452

**Published:** 2004-02

**Authors:** Tze-wai Wong, Chin-kei Lee, Wilson Tam, Joseph Tak-fai Lau, Tak-sun Yu, Siu-fai Lui, Paul K.S. Chan, Yuguo Li, Joseph S. Bresee, Joseph J.Y. Sung, Umesh D. Parashar, for the Outbreak Study Group

**Affiliations:** * The Chinese University of Hong Kong, Hong Kong Special Administrative Region (SAR), People’s Republic of China; † National Centre for Epidemiology and Population Health, Australian National University, Canberra, Australia; ‡ Hospital Authority, Hong Kong SAR, People’s Republic of China; § The University of Hong Kong, Hong Kong SAR, People’s Republic of China; ¶ Centers for Disease Control and Prevention, Atlanta, Georgia, USA

**Keywords:** severe acute respiratory syndrome, transmission, superspreader, Hong Kong, research

## Abstract

We studied transmission patterns of severe acute respiratory syndrome (SARS) among medical students exposed exclusively to the first SARS patient in the Prince of Wales Hospital in Hong Kong, before his illness was recognized. We conducted a retrospective cohort study of 66 medical students who visited the index patient’s ward, including 16 students with SARS and 50 healthy students. The risk of contracting SARS was sevenfold greater among students who definitely visited the index case’s cubicle than in those who did not (10/27 [41%] versus 1/20 [5%], relative risk [RR] 7.4; 95% confidence interval [CI] 1.0 to 53.3). Illness rates increased directly with proximity of exposure to the index case. However, four of eight students who were in the same cubicle, but were not within 1 m of the index case-patient, contracted SARS. Proximity to the index case-patient was associated with transmission, which is consistent with droplet spread. Transmission through fomites or small aerosols cannot be ruled out.

Severe acute respiratory syndrome (SARS) is a newly recognized clinical entity associated with infection by a novel coronavirus (SARS-CoV) (*1*–*4*). SARS is characterized by symptoms of fever, chills, headache, and dry cough, with radiographic evidence of pneumonia in most patients. The incubation period of SARS is estimated to be a median of 4 to 6 days (range 2–10 days). SARS is contagious, and person-to-person transmission appears to occur primarily through contact or respiratory droplets (*5*). However, because of the efficient transmission of SARS observed in some situations (*6*,*7*), concerns remain about the spread of SARS-CoV through other means, including small aerosols or contact with contaminated environmental surfaces.

The pandemic of SARS is believed to have originated in late 2002 in Guangdong Province, China (*5*). A SARS patient from this region, who had onset of illness on February 15, 2003, traveled to Hong Kong and may have infected several guests at the hotel where he resided during February 21–22. One of the affected hotel guests was a resident of Hong Kong; on February 24, he exhibited an illness characterized by fever, cough, runny nose, and malaise. His symptoms worsened over the next few days, leading to his hospitalization on March 4 at the Prince of Wales Hospital, a major teaching hospital of the Chinese University of Hong Kong. The cause of this patient’s illness was not recognized until March 10, when secondary cases of SARS were first reported among healthcare workers; specific infection control measures were then implemented.

Epidemiologic investigations indicate that this patient transmitted SARS to 47 healthcare workers on the ward to which he was admitted; the administration of a bronchodilator through a jet nebulizer was widely believed to have contributed to this dramatic pattern (*1*). SARS developed in all but one of the 16 nursing staff members on the ward and in all 6 ward physicians. The first patient with a secondary case of SARS, which presumably resulted from infection by this index patient, was not hospitalized until March 11. Therefore, the period from March 4 to 10 provided a risk window during which the factors that affected transmission of SARS among persons exposed exclusively to this index patient could be assessed.

Although several groups of healthcare workers were exposed to SARS, some groups (e.g., ward nurses and doctors) could not provide useful information because most were affected by SARS, and other groups (e.g., staff in the accident and emergency department) could not recall all of their exposures to the index patient. However, a group of medical students who visited the ward had limited, well-defined exposures that could be accurately recalled. These included 20 third-year medical students who performed a bedside clinical assessment in the ward on the mornings of March 6 and 7, supervised by a team of assessors from the university. Each student was assigned to examine specific patients in the ward during a 40-minute interval on 1 of the 2 days. The locations (bed numbers) of the patients assigned to each student were precisely known, as well as the relative location of these patients to the index SARS case-patient. In addition to the students who appeared for the assessments, several other students (mostly fifth-year students) visited the ward for bedside teaching or clinical training March 4–10. We analyzed the epidemiologic features and patterns of transmission of SARS among these students.

## Methods

### Study Population

We conducted a retrospective cohort study of medical students who visited the index patient’s ward from March 4 to March 10, 2003. To define the study cohort, all 474 medical students of the university who were in their clinical years (years 3–5) were contacted to inquire whether they had visited the patient’s ward during this period. Because the university classes were suspended in response to the outbreak at the time this investigation was begun, the students were contacted by electronic mail.

### Data Collection

Students who reported visiting the patient’s ward during the period were given a detailed questionnaire that sought information about demographic characteristics, history of recent illnesses, activities in the ward (including specific exposure to the index patient), use of personal protective equipment, and history of travel March 1–10. Students who contracted SARS were interviewed in the hospital wards where they were admitted. To facilitate the recall of exposures to the index patient, a map showing the location of the index patient on the ward was distributed with the survey. Survey responses were validated by a follow-up telephone interview or electronic mail communication. Data provided by students regarding the bed numbers of patients they examined during their bedside clinical assessment were cross-checked with the university records. The medical (including nursing) records of the index patient and the students who were ill with SARS were reviewed.

### Case Definition

A case of SARS was defined by the presence of fever (temperature >38°C) and evidence of pneumonia on either a radiograph or computed tomographic image of the thorax, with or without respiratory symptoms (e.g., cough and shortness of breath).

### Laboratory Studies

Paired serum specimens were obtained during the acute phase and convalescent phase (day 21 from onset of fever) of illness from ill students, and single serum samples were obtained during April 26 to May 3 from students who visited the ward during March 4 to 10 but did not acquire SARS. The serum specimens were tested for anti–SARS-CoV immunoglobulin (Ig) G by indirect immunofluorescence, by using SARS-CoV–infected Vero cells fixed in acetone. A positive test was defined as either seroconversion (>4-fold rise in antibody titer in the paired serum specimens) or a convalescent-phase antibody titer of >1:40.

### Ventilation Study

Information on the ward ventilation system was first obtained from the Electrical and Mechanical Services Department of the hospital. A detailed assessment of the ventilation system and airflow studies could not be performed at the time of the outbreak because of logistic constraints. Retrospective on-site inspections and measurements of the ventilation design and air distribution were carried out on July 17 and July 22. The supply and exhaust airflow rates were measured by a hood flow rate meter (APM 150) (TSI Inc., Shoreview, MN) (measurement range 24–945 L/s with an accuracy of 3%). Air velocity, air temperature, and relative humidity at all supply diffusers and exhaust grilles were measured by an portable VELOCICALC Plus air velocity meter Model 8386A (TSI Inc.). Information on the location and opening sizes of supply diffusers and exhaust grilles, as well as information on the distribution of heat sources such as lighting and the number of persons in the ward, were also collected during the site visits.

### Data Analysis

Epidemiologic data were entered into a predesigned database and analyzed by using SAS Version 6.12 software (SAS Institute Inc., Cary, NC). Attack rates among persons with and without specific exposures were calculated. Dose-response relationships were also evaluated with respect to the proximity to the index patient and duration of these exposures.

Data on ventilation, temperature, relative humidity, and heat sources were analyzed by computational fluid dynamics (CFD) simulations. The industry standard CFD package, Fluent, (Fluent USA, Lebanon, NH) was used to predict (reproduce) the average airflow pattern in the ward during the outbreak, taking into consideration the effect of thermal buoyancy.

## Results

### Clinical Course of the Index Patient’s Illness

On February 24, the index case-patient had onset of an illness characterized by fever, cough, runny nose, and malaise. His symptoms worsened over the next few days, and he sought treatment at the Accident and Emergency Department of the Prince of Wales Hospital on February 27, when he was treated as an outpatient and discharged. He visited the Accident and Emergency Department again on March 4 with the same symptoms and was admitted to a general medical ward. His fever (range 38°C–40°C) did not diminish after he received various antimicrobial drugs and persisted until March 11, when it gradually subsided. His cough was frequent, low-pitched, and unproductive, with occasional scanty, whitish sputum, and it persisted from March 4 to March 13; the cough was most severe during the first 4 days of his hospitalization, March 4–7. His chest radiograph on admission showed consolidation of the right upper lobe and patchy haziness in the right lower zone. He was weak, was given an intravenous drip, and remained bedridden during his first week of hospitalization. To relieve his respiratory symptoms, he was administered salbutamol through a jet nebulizer four times per day (at 10 a.m., 2 p.m., 6 p.m., and 10 p.m.) starting from 2 p.m. on March 6 until March 12, lasting about 30 min each time. His arterial oxygen on admission was 99%; it dropped to 95% on March 6, and gradually returned to 98% on March 12. He was identified as the index patient for the outbreak of SARS in Prince of Wales Hospital on March 12 and was transferred to an isolation room within the ward. He remained in isolation for 17 days after his symptoms subsided and was discharged on March 30. The patient was not treated with either ribavirin or steroids.

### Medical Student Study

Of the 474 medical students, 334 (70.5%) responded to the survey. Of the 334 respondents, 66 (20%) reported visiting the index patient’s ward during the study period. Respondents and nonrespondents did not differ in age and gender. SARS did not develop in any of the nonrespondents or in any of the respondents who did not visit the index patient’s ward. A detailed survey to assess illness and exposures was completed by these 66 students, which included the group of 20 third-year medical students who performed a bedside clinical assessment, supervised by a team of assessors from the university, in the ward on March 6 and 7, and 46 other students who visited the ward for clinical training on one or more occasions from March 4 to 10. None of the 20 students who appeared for the bedside clinical assessment visited this ward after March 7 or had any contact with other SARS patients in this hospital or in the community.

**Figure 1:**
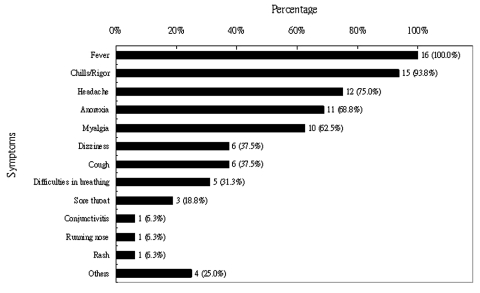
Distribution of initial symptoms in 16 students.

Sixteen (24%) of the 66 students reported an illness that met the case definition for SARS. Their mean age was 22.3 years, and 8 (50%) were male. The mean age of the 50 other students who visited the ward but did not acquire SARS was 23.2 years, and 23 (46%) were male. The most common symptoms of illness among the patients included fever (100%), chills or rigors (94%), and headache (75%); cough and shortness of breath were reported by 38% and 33% of patients, respectively (Figure 1). All ill students were hospitalized, and one required mechanical ventilation and treatment in the intensive care unit; all recovered from the illness. The characteristics of the illness among the students were similar to those among healthcare workers presumably infected by the index patient.

Paired serum specimens were collected from 15 of the 16 students during their illnesses, and all had demonstrable IgG antibodies to SARS-CoV at a titer of >1:40 in the convalescent-phase serum. The antibody titer ranged from 1:80 to 1:1,280, with a geometric mean titer of 1:440. Antibodies to SARS-CoV were absent in the serum specimens obtained from all 50 healthy students.

**Figure 2:**
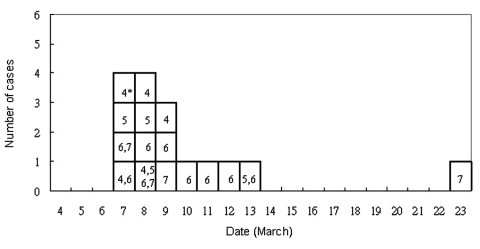
Dates of onset of illness of 16 students with severe acute respiratory syndrome and date of their visit to the index patient’s hospital ward. An asterisk indicates the dates of the visit in March 2003.

**Figure 3:**
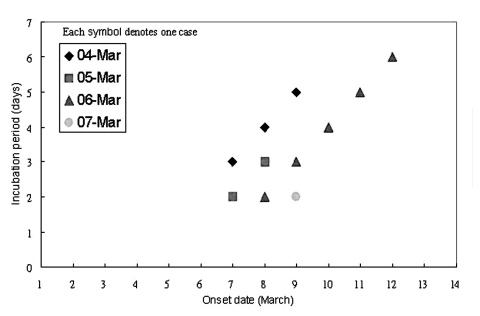
Incubation period by onset dates in 11 students.

The dates of onset of illness of the 16 students with SARS and the,[Carol: word is missing? Or comma shouldn’t be there?] dates they visited the ward are shown in Figure 2. The student with an unusually long incubation period of 16 days visited the ward (for a 40-minute bedside clinical assessment) on March 7. On March 13, she was noted to have pneumonic changes on a chest radiograph, although she had no symptoms. She was admitted to an observation ward for suspected SARS patients (different from the index patient’s ward) and was discharged on March 17 after resolution of her chest radiographic abnormalities. On March 23, fever developed, and she was readmitted as a potential SARS case-patient. Because we were not certain if this student had been infected during her initial exposure to the index case or during her subsequent hospitalization by exposure to another SARS patient in the observation ward, we excluded this student from the analyses of risk exposures. To obtain a precise estimate of the incubation period of SARS, we examined the onset of illness among 11 of the 16 ill students who visited the ward only on a single day, excluding the student with an incubation of 16 days. Among these 11 patients, the median incubation period was 3 days (range 2–6 days). Figure 3 shows the incubation period by onset date. Students exposed on March 6 had the widest range of incubation period (2–6 days). Too few students were exposed exclusively on other days to show any pattern.

**Figure 4:**
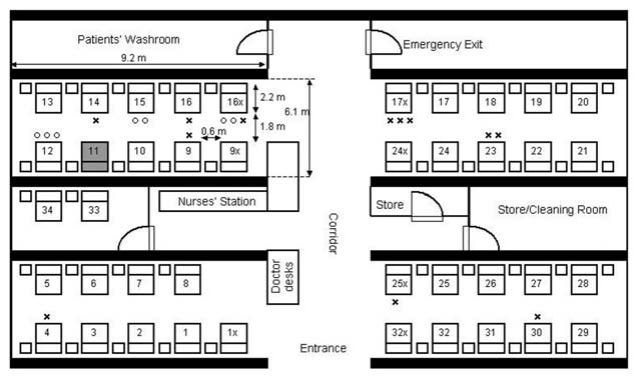
Floor plan of index patient’s hospital ward. Numbers with and without a suffix indicate the bed numbers of patients. The bed of the index patient is shaded. 0, students assigned to examine the patient in this bed who became ill with severe acute respiratory syndrome; x, students assigned to examine the patient in this bed who remained healthy.

We examined the attack rates of the illness among students based on whether they could recall entering the index patient’s cubicle, a semi-enclosed section of the ward containing 10 beds (Table 1). SARS developed in 10 of the 27 students who reported entering this cubicle, compared with SARS developing in 4 of the 18 students who could not accurately recall whether they entered the patient’s cubicle, and in only 1 of 20 students who reported that they never entered the cubicle (Mantel-Haenszel chi-square = 6.54; p = 0.011; Fisher exact test [2-tailed], p = 0.032). The student who did not enter the index patient’s cubicle but acquired SARS was a fifth-year student (not one of the third-year students who underwent the bedside clinical assessment) who reported visiting the patient in bed no. 17x, which was located in the opposite cubicle adjacent to the corridor (Figure 4). Among those students who could recall accurately whether they entered the patient’s cubicle, entering the cubicle was significantly associated with illness (10/27 versus 1/20, relative risk = 7.4, 95% confidence interval = 1.0 to 53.3, p = 0.046). The duration the students stayed in the ward was not associated with the risk for illness (mean length of stay: 67 minutes for the ill students; 80 minutes for the healthy students; p = 0.6).

**Table 1:** Attack rate for[?]of all students who visited the index patient’s cubicle in the ward

Entered index patient’s cubicle	Ill	Not ill	Total	Attack rate (%)^a^
Yes	10	17	27	37.0
Not sure	4	14	18	22.2
No	1	19	20	5.0
Total	15	50	65	23.1

^a^Fisher exact test (2-tailed), p = 0.032; Mantel-Haenszel chi-square = 6.54; p = 0.011.

To further assess the proximity of exposure associated with illness, we analyzed data from 19 of 20 medical students (excluding the ill student who had an unusually long incubation period) who appeared for the bedside clinical assessment (lasting 40 minutes for each student) on March 6 or 7. SARS developed in 7 of these 19 students. None of the students examined the index patient. All three students who examined patients located in beds within 1 m of the index patient contracted SARS; four of eight students who examined patients located in the same cubicle but in beds >1 m from the index patient contracted SARS, but none of eight student who examined patients in other cubicles fell ill (Mantel-Haenszel chi-square = 9.86, p = 0.002; Fisher exact test [2-tailed], p = 0.0031) (Table 2; Figure 4).

**Table 2:** Attack rate for students attending a bedside clinical assessment in the ward in relation to their proximity to the index patient’s bed^a,b^

Location of exposure	Cases/no. of students exposed
Bed nos. 10 and 12 (adjacent to index patient)	3/3
Bed nos. 9, 9x, and 13–16x (beds in the same cubicle except bed nos. 10–12)	4/8
Other beds in the ward (not in the cubicle)	0/8

^a^The index patient was not used as an assessment case. ^b^Mantel Haenszel chi-square = 9.86, p=0.002; Fisher exact test (2-tailed), p = 0.0031.

As mentioned previously, the index patient was administered nebulizer therapy four times per day starting from 2 p.m. on March 6 until March 12, lasting about 30 minutes each time. Among all the students, no significant association was noted between their risk for illness and presence in the ward when the nebulizer was in use. To further study the potential role of nebulizer therapy in disease transmission, we studied the temporal patterns of illness among these 19 students who appeared for a bedside clinical assessment, excluding the student with a long incubation period (Table 3). Six out of 10 students assessed on March 6 before the nebulizer was used contracted SARS compared with 1 out of 9 students on March 7. The time of assessment of the student with SARS (on March 7) coincided with the use of the nebulizer.

**Table 3:** Time schedule of the clinical assessment of 19 medical students^a^

Time		Ill/total
6 March 2003	10:00–10:40 a.m.	0/3
	10:40–11:20 a.m.	2/3
	11:30 a.m.–12:00 p.m.	3/3
	12:00–12:40 p.m.	1/1
7 March 2003	10:00–10:40 a.m.	1/2
	10:40–11:20 a.m.	0/3
	11:30 a.m.–12:00 p.m.	0/3
	12:00–12:40 p.m.	0/1

^a^Excluding the student-patient whose illness had a long incubation period.

The medical students were assessed by a total of 11 assessors. Five assessors evaluated students on March 6 only, five on March 7 only, and one was present on both days. SARS was reported by all five assessors for March 6 only, by three of five assessors for March 7 only, and by the one assessor who was present on both days.

None of the students had traveled to mainland China, the only location with suspected community transmission of SARS during the study period. None of the ill students reported contact with another ill student or other person with SARS in the 10 days before illness onset. None wore masks or gloves while examining patients, and no notable differences in risk for disease were observed among students who reported washing their hands before and after examining patients. Apart from one hepatitis B carrier (who contracted SARS), no other students had any chronic illness. The clinical course and severity of illness in the hepatitis B carrier were similar to the experiences of other students.

### Ventilation Study

#### Ventilation System

The hospital is centrally air-conditioned. Fresh air is drawn from outside the hospital building into a primary air unit situated in a room adjacent to the ward, where it is cooled by chilled water and then supplied to this ward (and another ward on the opposite side of the hospital) through air ducts. The air is then distributed to five fan-coil units (one in each of the four cubicles and one at the nurses’ station), where it is mixed with recirculated air, cooled by chilled water, and blown into the cubicle/nurses’ station via air supply diffusers (0.6 m by 0.6 m) located at the center of the cubicle in the false ceiling and over the nurses’ station. An exhaust grille, a rectangular opening 0.3 m by 0.6 m, located in the false ceiling in the corridor outside each cubicle and outside the nurses’ station, recirculates 70% of the air supply back into the fan-coil unit. Excess air escapes through two extraction fans inside the toilet, two extraction fans in the store/cleaning room, and through the door of the ward to the outside.

### Airflow Measurements

**Figure 5:**
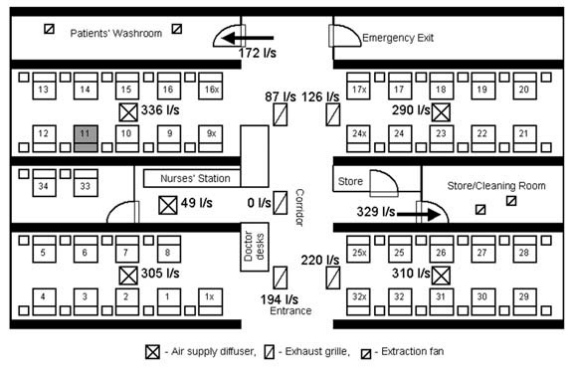
Airflow rates (L/s) through all air supply diffusers and exhaust grilles in the index patient's hospital ward. 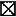, air supply diffuser; 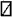, exhaust grille; 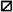, extraction fan.

The air exchange was 7.79 air changes per hour for the whole ward. The supply and exhaust airflow rates are summarized in Figure 5. The total air supply was higher than the total exhaust, which meant that the ward was at a positive pressure. Our on-site measurement showed that most of the extra air supply should have exited through the ward entrance because an exhaust fan was located in both the primary air unit room and the kitchen, just outside the entrance to the ward; these fans would create negative pressure.

The supply and exhaust airflow rates through diffusers and exhaust grilles were found to be imbalanced. The exhaust and air supply for the nursing station did not function properly. The air supply from the diffuser in the index patient’s cubicle had the highest supply flow rate (336 L/s), while the adjacent exhaust grille had the lowest exhaust flow rate (87 L/s) among all four functional exhaust grilles.

### Modeling the Dispersion of Hypothetical Aerosols

**Figure 6:**
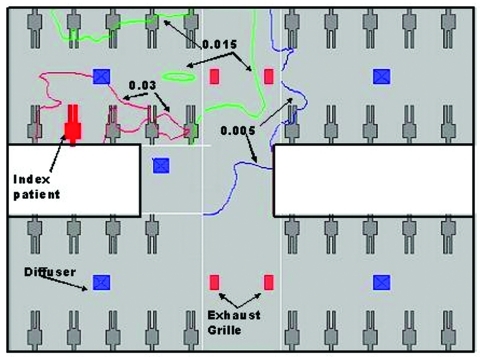
Dispersion of hypothetical aerosols that originated from the index patient’s bed in the ward. Three levels of normalized concentrations are shown (0.03, 0.015, and 0.05) because the source strength of the virus-laden aerosols is unknown.

At the time of the outbreak (March 4–10), the weather in Hong Kong was moderate with an ambient temperature ranging from 10.5°C to 22.3°C. The heat gains in the ward should be mainly from people, lighting, and equipment. In our computational fluid dynamics simulations to reproduce the average airflow pattern in the ward during the outbreak, we excluded the washroom and storeroom in our computational domain; and the exhaust flows through the two rooms were modeled as exhaust flows through their doorways. A free boundary condition was imposed on the ward entrance. Our computational fluid dynamics package could also consider the movement and evaporation of the aerosols. We found that aerosols would rapidly evaporate and the size of droplets would decrease rapidly after they originated from the index patient’s bed. The average air speed in the room was pproximatelyaround 0.2 m/s. The normalized concentration contours of hypothetical aerosols are shown in Figure 6. The concentrations decreased as we moved away from the index patient’s bed. We also predicted a fairly high concentration profiles for beds 17x and 24x in the opposite cubicle. The concentrations in other two cubicles were almost zero.

## Discussion

We utilized a unique opportunity provided by an unrecognized SARS patient who was the only known source of infection for a large cluster of secondary cases in an institutional setting to examine the transmission patterns of this novel disease. Proximity to the index case was associated with transmission, and all three students who examined the patient in bed 12 (within 1 m of the index patient) contracted SARS. As the index patient was bedridden during this period, this observation is compatible with transmission by droplets. However, that a few ill students were never within 1 m of the index patient raises the possibility of transmission by other mechanisms. Spread by contaminated fomites is a possibility, especially in light of recent data indicating that SARS-CoV survives well in the environment (*8*). Although none of the students reported direct contact with any of the index patient’s belongings or linen, contact with other articles in the ward contaminated by the patient’s secretions or body fluids might have occurred. Transmission by aerosols over a limited distance could also explain the observed distribution of cases and the large number of cases among healthcare workers on the ward. In our ventilation study, we found that the airflow rate was highest in the air supply diffuser in the index patient’s cubicle and lowest in the corresponding exhaust grille. This imbalance and the computed concentration contours of aerosols (which match our epidemiologic data) are compatible with spread by aerosols. However, because we were not able to conduct a detailed study of ventilation patterns or conduct environmental and air sampling at the height of the outbreak due to logistic constraints, we cannot definitively assess whether either fomites or aerosols played a role in transmitting virus from the index patient.

At the time this investigation was begun, jet nebulizer therapy given to the index patient was widely believed to have facilitated transmission. However, our findings demonstrate efficient transmission even before nebulizer therapy was begun on the afternoon of March 6. First, 6 of the 10 students who attended the bedside clinical assessment on the morning of March 6 contracted SARS, compared with 1 of the 9 who attended the assessment on March 7. Second, all five of the assessors who assessed students on March 6 alone became ill, compared with three of the five assessors who were present on March 7 alone. Lastly, for the students with SARS who were present on the ward for reasons other than the bedside assessment, no association was observed between their stay in the ward at the specific periods when the nebulizer was used and the development of SARS. However, because nebulizer therapy could theoretically exacerbate symptoms of coughing in SARS patients, we recommend avoiding the use of nebulized medications and other potential aerosol-generating patient-care procedures if possible and using appropriate infection control precautions if such procedures are deemed necessary (*9*).

Similar large “superspreading events” of SARS associated with a single patient have been described in several countries (*5*,*6*), which contrast with the limited secondary spread seen with most SARS patients. Because many of the index patients in these clusters were infected with early cases of SARS in their respective countries, such as the index patient for this outbreak, or had subtle or atypical manifestations, the failure to recognize the disease early and institute appropriate infection control precautions might have contributed to extensive transmission. Also, some SARS patients may be intrinsically more contagious. They might excrete greater amounts of virus in their secretions or transmit virus by different routes, which may be related to specific host (e.g., altered immune status, underlying diseases), agent (e.g., coinfections with other pathogens), or environmental factors that require further study. Superspreading events have been reported in outbreaks of other diseases such as Ebola hemorrhagic fever, rubella, and β-hemolytic streptococci (*10**–**12*)*.* While the mechanisms for these phenomena are largely unknown, possible explanations include a larger number of contacts of these superspreaders, inherent differences in the virus-host relationship, or the presence of a more virulent strain or higher levels of virus shedding (*10*). Similarly, hospitals have previously been documented as settings for efficient transmission of illnesses such as Lassa fever and Bolivian hemorrhagic fever (*13*,*14*).

In conclusion, this cluster demonstrates the potential for widespread nosocomial spread of SARS among a previously healthy population in the absence of specific infection control precautions. SARS is likely spread through direct contact and respiratory droplets in most instances, and others have demonstrated that specific infection control precautions to prevent transmission by these mechanisms are effective (1*5*). However, we cannot exclude the role of contaminated fomites or small aerosols in transmitting virus in this outbreak. Whether this large cluster resulted fromof different mechanisms of transmission, greater viral shedding by the patient, or inadequate infection-control measures is not known, but it clearly indicates that SARS can be spread highly efficiently in some situations. A better understanding of the phenomenon of superspreading events, including clusters with apparently unique patterns (*15*), is key to assessing the pandemic potential of SARS and the effectiveness of control measures (*16*,*17*).
